# Assessment of biological hazards in a laboratory in Boa Vista, Roraima

**DOI:** 10.47626/1679-4435-2023-818

**Published:** 2023-04-18

**Authors:** Aline Farias Ribeiro, Maria Vivina Barros Monteiro, Reinaldo Eduardo Costa Silva-Júnior, René Ribeiro Silva

**Affiliations:** 1 Programa de Pós-Graduação em Doenças Tropicais, Universidade Federal do Pará (UFPA), Belém, PA, Brazil; 2 Programa de Pós-Graduação em Análises Clínicas, UFPA, Belém, PA, Brazil; 3 Hospital Geral de Roraima, Roraima State Government, Belém, PA, Brazil

**Keywords:** health care, exposure, hazard, saúde, exposição, risco

## Abstract

**Introduction:**

Safety in health care should be a priority of all health professionals. The occurrence of occupational accidents is many times attributed to a failure in following established standards, and identifying and correcting the risks to which professionals are exposed is important.

**Objectives:**

The aim of this study was to assess the level of understanding on the biological hazards to which workers of a clinical analysis laboratory are exposed.

**Methods:**

We applied a questionnaire for assessing knowledge on biological hazards, comprising an assessment of the understanding of biosafety and biological hazards, an investigation of the occurrence, types, and causes of accidents with biological material, and the employment of preventive measures. Data were tabulated in spreadsheets. All qualitative variables were tested with the chi-square test.

**Results:**

We verified that 100% of the workers reported having some knowledge on biosafety; 25% of them reported they had suffered an occupational accident; and 81% of the workers reported having received training on biosafety measures. As to the level of exposure of workers and the community to biological agents, we noticed a very low level of exposure in one of the laboratory sectors.

**Conclusions:**

Considering our results, we concluded that professionals at a clinical analysis laboratory are prone to occupational hazards, facing a low risk of exposure despite carrying out hazardous activities with potential exposure, which requires caution and exposure prevention measures.

## Abstract (pt)

**Introdução:**

A segurança na assistência médica deve ser uma prioridade de todos os profissionais da área da saúde. Muitas vezes, a ocorrência de acidentes de trabalho é atribuída ao não seguimento dos padrões definidos, sendo importante identificar e corrigir os riscos a que os profissionais estão expostos.

**Objetivos:**

O objetivo deste estudo foi avaliar o nível de compreensão sobre os riscos biológicos a que os trabalhadores de um laboratório de análises clínicas estão expostos.

**Métodos:**

Foi aplicado um questionário para avaliação sobre o conhecimento dos riscos biológicos, composto por avaliação sobre o conhecimento dos conceitos de biossegurança e riscos biológicos; investigação da ocorrência, dos tipos e das causas de acidentes com materiais biológicos; e uso de medidas preventivas. Os dados foram tabulados em planilhas. Todas as variáveis qualitativas foram testadas usando o teste do qui-quadrado.

**Resultados:**

Verificou-se que 100% dos trabalhadores relataram que possuem conhecimento sobre biossegurança; 25% relataram já ter sofrido algum acidente em serviço; e 81% dos trabalhadores relataram que receberam treinamento sobre as medidas de biossegurança. Quanto ao nível de exposição dos trabalhadores e da comunidade aos agentes biológicos, constatou-se um nível de exposição muito baixo em um dos setores do laboratório.

**Conclusões:**

Diante do exposto, conclui-se que os profissionais de laboratório de análises clínicas têm predisposição aos riscos ocupacionais, enfrentando um risco baixo de exposição apesar de desempenharem atividades insalubres e com potencial de exposição, o que requer cuidados e medidas de prevenção à exposição.

## INTRODUCTION

Safety in health care should be a priority of all health professionals. Health care employers and workers should provide quality care to patients in an environment that is safe for all. Creating a safe workplace is not optional; instead, it is required by law.^1^

Each laboratory must have an updated procedure manual with specific safety guidelines. This document should be kept in a place accessible to anyone. The manual should include standard operating procedures (SOPs) and rules for the safe handling, storing, and disposal of chemical substances, in addition to strategies to be followed in case of fire. This manual should also provide safety rules and guidelines for training employees. The employer, supervisor, and instructor are responsible for monitoring the agreement and adherence of employees to safety rules and standards, thus avoiding risks. The adopted safety standards should be in accordance with the institution’s guidelines and regulations.^1^

Professionals at clinical laboratories face health hazards that can be classified into five categories: physical, chemical, biological, ergonomic, and accidents.^2^

Health professionals are at a higher risk of exposure to certain infectious diseases that are transmissible via respiratory and fecal-oral routes, as well as contact with blood and other body fluids. Occupational exposure to biological material represents a risk to workers at clinical laboratories due to the possibility of transmitting pathogens such as hepatitis B and C viruses (HBV and HCV) and HIV. The consequences of this exposure may affect workers directly, including their physical, psychological, family, and social aspects.^3,4^

Occupational accidents with exposure to biological material involve blood and other body fluids and happen with health care workers on the job, where they are exposed to potentially contaminated biological material.^5^ The occurrence of occupational accidents is many times attributed to a failure in following established standards, wearing personal protective equipment (PPE), among other factors. However, many other variables contribute to the occurrence of failures, such as lack of training; inexperience; lack of safety equipment; fatigue; repetitive tasks; double burden; emotional disorders; excessive self-confidence; low professional qualification; work disorganization; lack of mental preparedness in emergency situations; negligence; lack of a clear definition of the institution’s quality standards, workflows, and responsibilities; and lack of periodic training and audits.^6^

**Figure 1:**
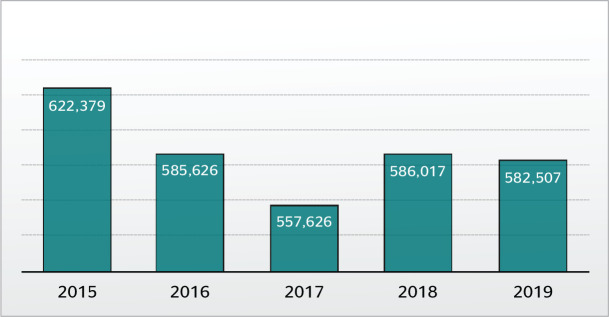
Occupational accidents recorded in Brazil from 2015 to 2019. Source: Social Security.^7^

**Figure 2:**
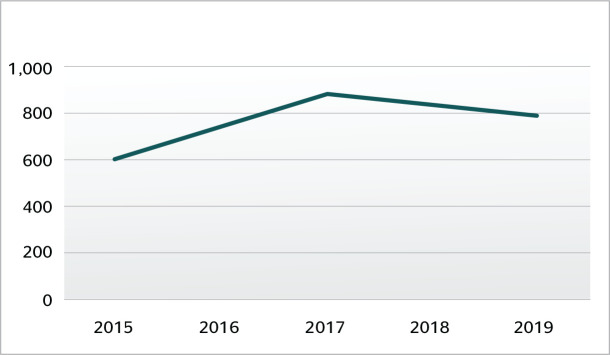
Occupational accidents recorded in Roraima from 2015 to 2019. Source: Social Security.^7^

Statistical data for occupational accidents in 2019, as published by the Ministry of Social Security, indicate a slight decrease in the number of occupational accidents recorded in Brazil in comparison with previous years (Figure 1), but a slight increase in the state of Roraima (Figure 2). In 2019, 582,507 accidents were reported in Brazil; of these, 795 were in Roraima.^7^

The Occupational Safety and Health Administration (OSHA) was implemented by the United States Congress and establishes standards to reduce diseases, accidents, and deaths at the workplace. The Centers for Disease Control and Prevention (CDC) and OSHA, as well as other United States government agencies, led the development of safety guidelines (universal precautions, UPs) for protecting health professionals and the public against infectious agents. The adopted precautions include the use of PPE such as lab coats, gloves, masks, and eye protection gear.^1^

It is important to identify the risks so that actions can be preemptively taken, avoiding potential failures or damages. The NBR ISO 31,000/2009 standard, titled “Risk management - Principles and guidelines,” by the Brazilian Association of Technical Standards (ABNT), guides and facilitates this identification in its assessment of prioritization in decision-making, enabling the implementation of strategies to improve laboratory risk management.^8^

The management system approach allows an organization/company to identify, monitor, and control laboratory biosafety and biosecurity aspects of its activities, effectively approaching the management system. This approach should be constructed based on the concept of continuous improvement of a cycle involving the planning, implementation, review, and improvement of the processes and actions an organization commits to, also known as the Plan-Do-Check-Act (PDCA) principle: in order to improve the management of biohazards by focusing on the causes of noncompliances and undesirable events, systematically identifying and correcting flaws in the performance system and biohazard control.^9^

In Brazil, there are various recommendations by the International Labour Organization (ILO) for regulating this area; these are ratified by Ministry of Labor directives named Regulatory Standards (NRs), in addition to the Consolidation of Labor Laws (CLT). Studies on occupational hazards state that, when uncontrolled, hazards lead to accidents and occupational diseases. The Ministry of Labor, via the NRs, aims to eliminate or control these hazards.^10^

Starting from the principle that clinical analysis laboratories - establishments dedicated to collecting and processing human material in order to perform laboratory tests and examinations - concentrate a series of hazards that can lead to health problems in workers, enumerating hazards becomes important to identify them and assess the risks to which workers and service providers working at these sites are subjected. As a consequence, hazards at the laboratory are multidimensional from the viewpoint of both the stability and previsibility of results. Risk management necessarily involves all levels of the company.^11^ Therefore, being able to identify and correct these risks is vital to the safety, effectiveness, and efficiency of services provided by the institution and to the society as a whole, since noncompliance could cause irreparable harm to everyone involved.

The aim of this study was to assess the level of understanding about biological hazards at a clinical analysis laboratory (microbiology, hematology, urinalysis, and biochemistry) in the city of Boa Vista, state of Roraima.

## METHODS

This study was approved by the Research Ethics Committee of Hospital Universitário João de Barros Barreto, opinion No. 2,095,053, and was performed in 2018. This study included 31 workers of a hospital in Roraima, whose activities were focused on the technical area (laboratory technicians, biomedical scientists, pharmacists). We applied a questionnaire with questions for assessing knowledge about biological hazards under supervision of an investigator. The questionnaire was divided into three parts: part 1 - assessment of knowledge on biosafety and biological hazards to which workers are exposed; part 2 - investigation of the occurrence, types, and causes of accidents with biological material; and part 3 - employment of preventive measures.

For assessing the level of exposure to biological hazards, we applied a questionnaire to an employee of one of the laboratory sectors using Biosafety Assessment Model (BIORAM) software, version 1.0; risks were categorized as very high, high, moderate, low, and very low. Data were tabulated in Microsoft Excel 2010 spreadsheets. All qualitative variables were tested using a chi-square test for comparing the observed and expected proportions. Affirmative answers were tested as to the degree of agreement between answers by the Kappa test. The significance probability was set at 0.05.

## RESULTS

In this study, most participants were female (72%), and participants of both sexes were aged between 27 and 62 years; most participants were aged between 30 and 39 years (57%).

As to their job tenure, 9 (28%) participants reported working at the laboratory for less than 1 year, 14 (43%), reported working for 1 to 4 years, and 4 (12%) reported working at the institution for more than 7 years.

### ASSESSMENT OF BIOSAFETY CONCEPTS

We verified that 100% (31) of the workers reported having knowledge on biosafety, and 29 (93.5%) had done courses on the theme; all participants correctly defined the term “biosafety.”

As to the definition of “hazard,” approximately 47% (15) of the workers answered it correctly, and 19% (6) did not answer the question even though all workers reported they were aware of the risks inherent to their profession and most of them (93%) reported that biological, ergonomic, chemical, and physical hazards, as well as the risk of accidents, fit their exposure the most. Of these, the manipulation of body fluids, secretions, and excretions was reported by all the study participants as the greatest source of exposure. All the study participants correctly defined PPE.

### INVESTIGATION OF THE OCCURRENCE, TYPES, AND CAUSES OF ACCIDENTS WITH BIOLOGICAL MATERIAL

Regarding the occurrence of accidents at their workplace, 25% (7) of the workers reported they had suffered an accident on the job; of these, 75% reported accidents involving biological material and 25% reported sharps accidents. Among accident causes, workers reported factors related to the institution (lack of equipment maintenance, wearing wrong size gloves, reusing glassware) and to the worker (lack of PPE use, performing a repetitive activity, hurrying, and lack of attention). No association was observed between training on biosafety and suffering an occupational accident; therefore, these variables were independent (p = 0.425).

Among those who suffered accidents, 50% reported that they informed someone at the institution of the accident, but only 37.5% notified the accident and 50% did not seek medical assistance. The reasons presented for not seeking medical assistance were the nature of the accident (superficial), the lack of another employee for covering their position, and superficial spillage.

### EMPLOYMENT OF PREVENTIVE MEASURES

Approximately 81% (26) of workers reported having received training on biosafety measures. Of these, 62.5% received it before starting their activities at the institution and 37.5% received training after starting their activities.

Most workers (65.6%) reported the use of collective protective equipment (CPE), of which the biological safety cabinet was the most commonly reported. As to the use of PPE, all interviewees stated that they used lab coats, gloves, and masks during their work activities, and 87.5% reported that PPE were provided by the institution. However, 72% had acquired some piece of PPE themselves for performing their work activities. At statistical analysis, no association was seen between receiving training and use of PPE by employees (p = 0.509).

Around 12.5% (4) of the interviewees did not know how to proceed in case of an accident with biological material, and 25% (8) did not know what a hazard map was. All professionals reported they were vaccinated with most available vaccines (measles, mumps, and rubella [MMR], HCV, yellow fever, influenza, measles, whooping cough, bacille Calmette-Guérin [BGC]).

### ASSESSMENT OF THE LEVEL OF EXPOSURE TO BIOLOGICAL HAZARDS AT THE LABORATORY

**Figure 3:**
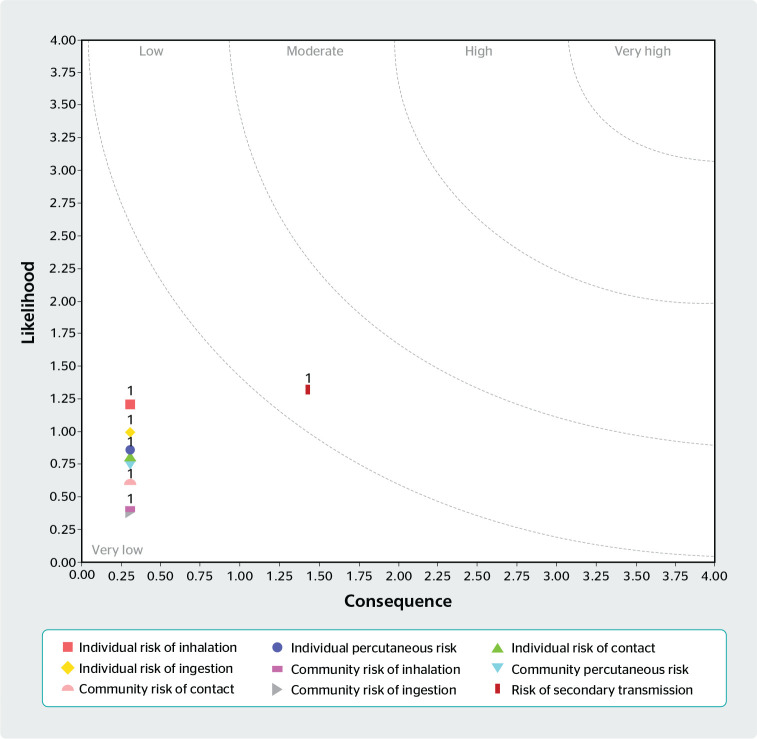
Biosafety hazards for each agent considering the individuals and the community at the laboratory. Source: Biosafety Assessment Model (BIORAM) software.

As to the level of exposure of workers and the community to biological agents manipulated at a laboratory sector, we noticed a very low level of exposure for the following risks: inhalation, ingestion, percutaneous, and contact. However, a higher risk of exposure was observed for secondary transmission when compared to the others, as demonstrated in Figure 3.

## DISCUSSION

In a hospital environment, health professionals are exposed to various risks. This workplace is thus considered hazardous, as it puts professionals at risk of various occupational hazards.^12^

Since professionals at a clinical analysis laboratory remain longer periods in a hazardous environment, carrying out procedures with biological samples and in direct contact with patients, they are more exposed to the occupational hazards of this workplace.

In this study, 100% of the workers reported knowing the term “biosafety,” which was confirmed by inquiring about its meaning; all professionals answered the question correctly. Despite having some knowledge on biosafety, less than half (47%) of the workers were aware of the hazards they were exposed to, mentioning biological hazards as the most prevalent. A review by Monteiro et al.^13^ reported that biological hazards are very frequent in the routine of health professionals, leading to a risk of contamination by the professional and a risk of transmitting infectious agents to patients, possibly causing diseases transmitted through percutaneous exposure. This demonstrates the importance of knowing the hazards inherent to these professionals so that measures for minimizing this exposure can be taken.

Bernardo^14^ reported that laboratory accidents mainly occur due to intrinsic errors such as personal beliefs, values, and behaviors. In this study, 25% of the workers reported having suffered some kind of accident on the job, and no association was verified between those who suffered accidents and lack of training. This can corroborate the previously mentioned study,^14^ as our participants reported lack of equipment maintenance, reusing glassware, lack of PPE, hurrying, and lack of attention during tasks as possible causes of accidents. Considering this situation, the creation of educational actions on good laboratory practices is required in order to raise awareness among laboratory professionals for adopting these measures.

A study performed by Gessner et al.^15^ observed that current working conditions, characterized by a lack of employees and task overload, hinder or block investments in occupational health such as permanent education. The study shows that the absence of training leaves implies in worker exposure, since no guidance on health protection is provided; this also applies to workers in our study due to the low portion (37.5%) of accident notifications done by professionals, where some were not reported due to lack of employees for taking over their duties. Occupational accident notifications should be done using dedicated forms, standardized by the Ministry of Health, at the Information System on Diseases of Compulsory Declaration and specific sentinel networks, with the aim of executing prevention and control policies for these accidents.^16^ In view of these results, it is vital that laboratory workers are instructed as to the importance of notifying accidents so that measures that protect workers’ health and minimize risks inherent to the accident can be taken. The notification allows the diagnosis of an event by understanding the causes and determinant factors of work that may contribute to identifying a sector’s epidemiology, since accidents are avoidable and predictable.^16^

As to the employment of preventive measures, most workers reported having received training before starting their activities. Some studies^13,15,17-21^ ratified the importance of training health professionals for the adequate performance of their activities and to decrease risks inherent to the profession. We thus verified that the study participants were trained as stated by the current legislation, although a share of the interviewees (12.5%) did not know how to proceed in case of an accident with biological material. This highlights the need for continuous training and assessments of training effectiveness, as well as the adoption of a periodic review of SOPs and manuals that rule laboratory good practices.

Most participants reported the use of biological safety cabinets as CPE, and considering the use of PPE, all professionals reported using lab coats, gloves, and masks. We noticed that 72% of the professionals had acquired some piece of PPE on their own for performing their work activities, leading to the conclusion that PPE is provided by the institution, but in inadequate amounts for the execution of the workers’ activities. The use of PPE and CPE is essential for preventing occupational accidents and diseases, especially when considering laboratory professionals, as they are susceptible to occupational hazards due to contact with biological samples, sharps, reagents, and equipment, in addition to a variety of disease-causing pathogens.^22^ The equipment minimizes the consequences of accidents, since it reduces the possibility of harming the individual.^23^ The institution must thus provide these items in sufficient amounts for professionals to perform their activities, reinforce the need to wear them, and hold employees accountable for not wearing them in order to avoid exposure to these risks.

In this study, 25% of the participants did not know what a hazard map was, and the investigator did not identify a hazard map on site at the moment the questionnaire was applied to the workers. This instrument is of vital importance for illustrating the hazards to which professionals are exposed, with the aim of contributing to accident prevention in that environment.^13,24^ Therefore, the institution must create hazard maps for each laboratory environment. According to Da Silva & Valente,^24^ this should be performed with the presence of professionals specialized in the area to facilitate risk identification, enable higher interaction among the team, and stimulate the participation of everyone in accident prevention actions.

Among preventive measures considering hazard exposure, we cite the vaccination of professionals against certain diseases, such as HBV, influenza, and tuberculosis. All professionals in this study reported being vaccinated according to the immunization schedule adopted by the Ministry of Health and Regulatory Standard No. 32 of the Ministry of Labor and Employment. Vaccines should be provided to workers at health care services free of charge and when there are effective vaccines against biological agents to which they may be exposed.^25^

According to the Ministry of Health Protocol for Exposure to Biological Material,^3^ exposure to biological material should be assessed considering its transmission potential based on the following criteria: type of exposure; type and amount of fluid and tissue, serological status of the source; serological status of the person who suffered the accident, and susceptibility of the exposed professional. The type of exposure can be percutaneous, mucosal, and non-intact skin. When assessing the type of exposure to biological hazards in a laboratory sector in this study, we verified that, for most risks (inhalation, ingestion, percutaneous, and contact), the activities performed at the laboratory presented a very low risk both to the individual and the community. However, when it comes to secondary transmission (that is, the transfer of etiological agents through animate or inanimate objects leading to disease spread), we verified a higher, but still low risk using BioRAM software. Therefore, laboratory professionals, despite carrying out hazardous activities in this sector with potential exposure to biological hazards, do not seem to face a high or moderate risk of exposure. It should be noted that although the activities performed at the laboratory do not pose a higher risk, the institution and its professionals should not overlook the risk prevention and mitigation measures. We highlight that this study was carried out in 2018 and, at that moment, professionals did not manipulate microorganisms with pandemic potential such as what we are going through with the global health emergency caused by COVID-19.

This study calls for attention to the need for constant training of health professionals, especially those at clinical analysis laboratories, who work in hazardous conditions with continuous exposure to various occupational hazards that may affect their life conditions. Moreover, it also demonstrates the need for adopting preventive measures for minimizing the risks to which workers are exposed.

## CONCLUSIONS

This study came to the following main conclusions: a) professionals at clinical analysis laboratories are prone to occupational hazards in their workplace; b) less than half of the study professionals was aware of the risks they were exposed to; c) the factors involved in cases of occupational accidents were not related to a lack of training, but to intrinsic factors; d) occupational accidents were underreported by the employees during the study; e) workers have little knowledge on how to proceed in case of accidents; f) all professionals wore PPE for performing their tasks; g) a small fraction of the professionals did not know what was a hazard map; h) workers employ preventive vaccination measures for minimizing the risks to which they are exposed; and i) professionals of a laboratory sector, despite performing hazardous activities with potential exposure to biological hazards, face a risk of exposure when considering their usual situations.
